# Crystal structure of di-μ-acetato-di­acetatobis­(μ-6,6′-dimeth­oxy-2,2′-{[(propane-1,3-diylbis(aza­nylyl­idene)]bis­(methanylyl­idene)}diphenolato)tetra­zinc

**DOI:** 10.1107/S2056989015020551

**Published:** 2015-11-11

**Authors:** Xue Cai, Hui Ning

**Affiliations:** a Department of Chemistry, Mudanjiang Normal University, Mudanjiang 157011, People’s Republic of China

**Keywords:** crystal structure, zinc, Schiff base, acetate

## Abstract

The tetra­nuclear title complex, [Zn_4_(C_19_H_20_N_2_O_4_)_2_(CH_3_COO)_4_], is formed from two dinuclear motifs related by an inversion centre. The two crystallographically independent Zn^II^ ions in the asymmetric unit are in different coordination environments. One is square-based pyramidal with one O atom of an acetate group occupying the axial position and two N and O atoms of one bmspd [H_2_bmspd = *N*,*N*′-bis­(3-meth­oxy­salicyl­idene)propyl­ene-1,3-di­amine] Schiff base ligand forming the basal plane. The other Zn^II^ atom is six-coordinated by four O atoms of the bmspd ligand forming the equatoral plane and two O atoms of different acetate groups located in the axial positions. As a result, the two phenolic planes of the bicompartmental Schiff base ligand are distorted slightly. However, the planes of the two Schiff base ligands are parallel. In addition, the Zn—N and Zn—O bond lengths span the reasonable ranges 2.062 (2)–2.073 (2) and 1.9261 (15)–2.4356 (16) Å, respectively. The Zn⋯Zn distances separated by phenolic O atoms are 3.2466 (4) Å while the Zn⋯Zn distances bridged by acetate groups are 5.9835 (6) Å. The tetra­nuclear moieties are connected by van der Waals interactions, and form a chain along *c* axis.

## Related literature   

Metal-organic coordination complexes of *N*,*N*′-bis­(salicyl­idene)ethyl­enedi­amine (salen) Schiff-base derivatives have been studied extensively within the fields of homogeneous catalysis (Wezenberg & Kleij, 2008 ▸), non-linear optics (Rigamonti *et al.*, 2006 ▸), magnetics (Yuan *et al.*, 2007 ▸) and biological metalloenzyme mimics (Laskin *et al.*, 2008 ▸).
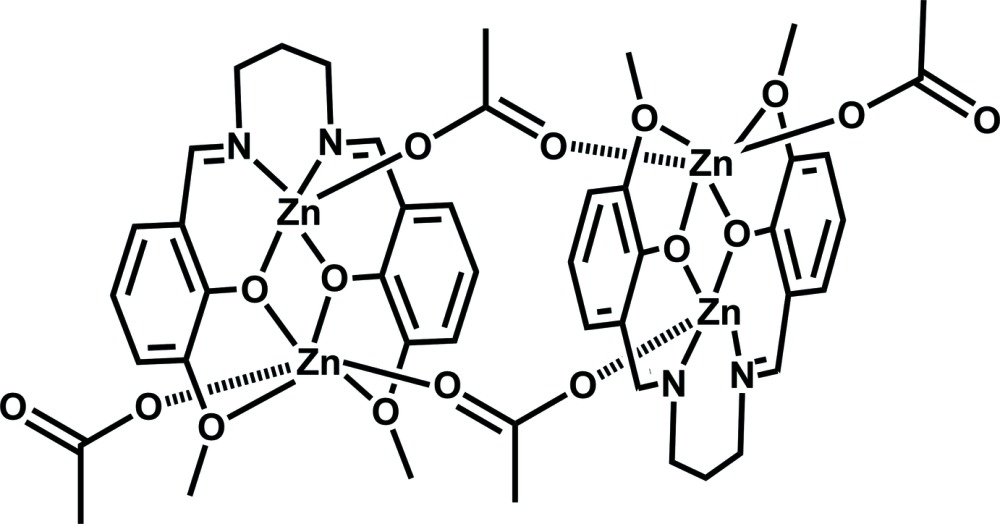


## Experimental   

### Crystal data  

[Zn_4_(C_19_H_20_N_2_O_4_)_2_(C_2_H_3_O_2_)_4_]
*M* *_r_* = 1178.48
Triclinic, 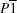

*a* = 10.4894 (9) Å
*b* = 10.7917 (9) Å
*c* = 11.9550 (11) Å
α = 103.425 (2)°
β = 94.323 (1)°
γ = 115.677 (1)°
*V* = 1162.17 (18) Å^3^
*Z* = 1
Mo *K*α radiation
μ = 2.12 mm^−1^
*T* = 298 K
0.15 × 0.10 × 0.08 mm

### Data collection  

Bruker SMART APEX CCD area-detector diffractometer
Absorption correction: multi-scan (*SADABS*; Sheldrick, 1996 ▸) *T*_min_ = 0.742, *T*_max_ = 0.849
6281 measured reflections
4497 independent reflections
3969 reflections with *I* > 2σ(*I*)
*R*_int_ = 0.013

### Refinement  

*R*[*F* ^2^ > 2σ(*F* ^2^)] = 0.024
*wR*(*F* ^2^) = 0.071
*S* = 1.05
4497 reflections
318 parameters
H-atom parameters constrained
Δρ_max_ = 0.26 e Å^−3^
Δρ_min_ = −0.27 e Å^−3^

### 

Data collection: *APEX2* (Bruker, 2004 ▸); cell refinement: *SAINT-Plus* (Bruker, 2001 ▸); data reduction: *SAINT-Plus*; program(s) used to solve structure: *SHELXTL* (Sheldrick, 2008 ▸); program(s) used to refine structure: *SHELXL97* (Sheldrick, 2008 ▸); molecular graphics: *XP* in *SHELXTL*; software used to prepare material for publication: *SHELXTL*.

## Supplementary Material

Crystal structure: contains datablock(s) 1, I. DOI: 10.1107/S2056989015020551/pj2024sup1.cif

Structure factors: contains datablock(s) I. DOI: 10.1107/S2056989015020551/pj2024Isup2.hkl

**Fig. 1.**
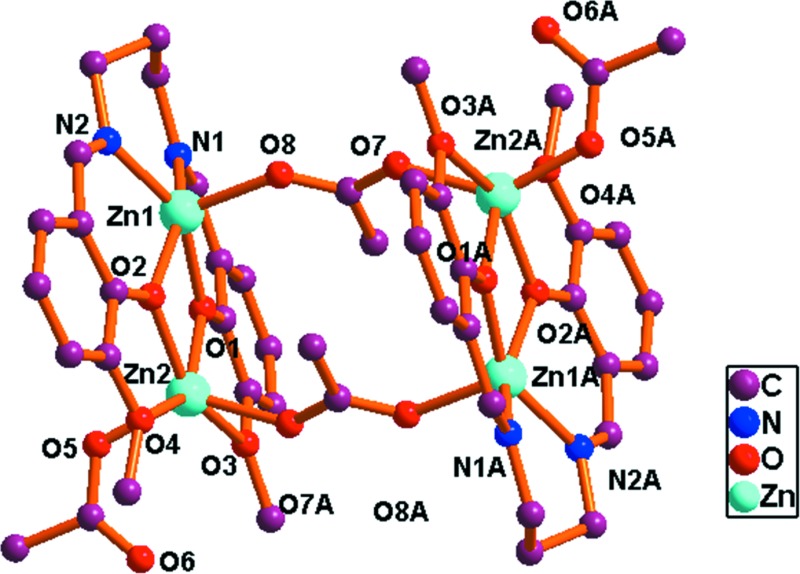
ORTEP diagram of molecular structure for complex [Zn4(bmspd)2(OAc)4] with atoms drawn as 50% probability ellipsoids.

ORTEP 4 2 4 . DOI: 10.1107/S2056989015020551/pj2024fig1.tif
*ORTEP* diagram of mol­ecular structure for complex [Zn_4_(bmspd)_2_(OAc)_4_] with atoms drawn as 50% probability ellipsoids.

**Fig. 2.**
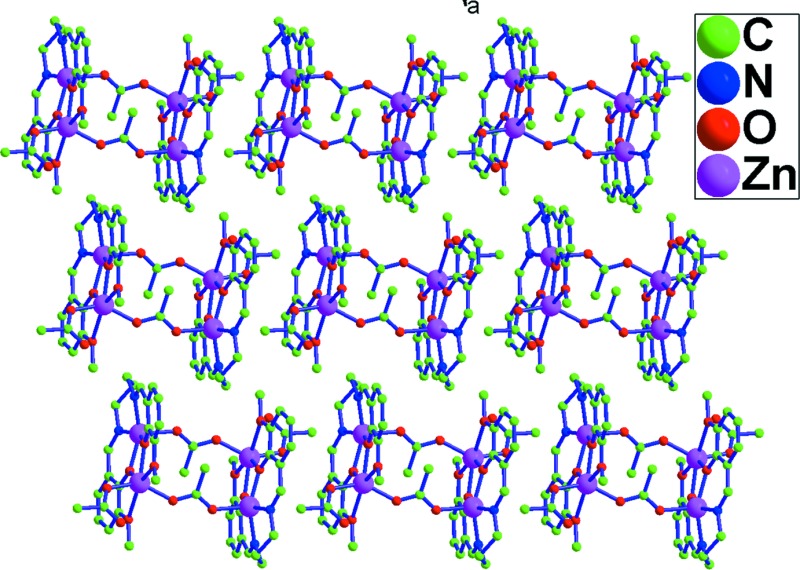
The perspective drawing of complex [Zn4(bmspd)2(OAc)4] packing in ac plane.

4 2 4 ac . DOI: 10.1107/S2056989015020551/pj2024fig2.tif
The perspective drawing of complex [Zn_4_(bmspd)_2_(OAc)_4_] packing in *ac* plane.

CCDC reference: 724776

Additional supporting information:  crystallographic information; 3D view; checkCIF report

## supplementary crystallographic information

### S1. Comment

The metal-organic coordination complexes of *N*,*N*'-bis(salicylidene)ethylenediamine (salen) Schiff-base derivatives were extensively studied within the field of homogeneous catalysis (Wezenberg *et al.*, 2008), nonlinear optic (Rigamonti *et al.*, 2006), magnetics (Yuan *et al.*, 2007), and biological metalloenzymes mimic (Laskin *et al.*, 2008). Recently, the compartmental salen ligands derived from the 2:1 condensation between 3-methoxysalicylaldehyde and corresponding diamines allowing for two metal ions located in dissimilar N_2_O_2_ and O_4_ cavities bridged by phenolic oxygen atoms, which bring interesting magnetic phenomena and intriguing optical properties. Herein, a novel heterometallic tetranuclear (Zn)_4_ compound has been obtained by step-by-step method and its structure is described.

The compound is composed of two [Zn_2_(bmspd)(OAc))]^+^ ions bridged by two acetate ions. There are two kinds of symmetry-independent zinc ions in this compound. One zinc ion (Zn1) is located in a five-coordinated environment, as shown in Figure 1. The other kind of zinc ion (Zn2) is coordinated by the O_4_ cavity in bmspd ligand and two oxygen atoms of acetate ions in pseudo-octahedral geometry. The neighbouring tetranuclear molecules form a two-dimensional supramolecular network by virtue of intermolecular π-π interactions. (Figure 2)

### S2. Experimental

A mixture of metal-free Schiff-base ligand H_2_bmspd (0.4 mmol 0.1368 g) and Zn(OAc)_2_·2H_2_O (0.4 mmol 0.0878 g) in methanol (20 ml) was stirred for 30 min at room temperature. Then, a solution of Zn(OAc)_2_·2H_2_O (0.4 mmol 0.0878 g) in methanol was added dropwise and the mixture was kept stirring for another 30 min at room temperature. Pale yellow block-shaped crystals for suitable for X-ray diffraction were obtained by slowly diffusing diethyl ether into the filtrate. The final mass of product was 0.0966 g.

### S3. Refinement

The coordinates of the difference H atoms bound to C atoms were placed using the HFIX commands in *SHELXL-97*, with C—H distances of 0.93–0.97 Å. All H atoms were allowed for as riding atoms with *U*_iso_(H) = 1.2*U*_eq_(C) or 1.5*U*_eq_(C). The orientation of hydrogen atoms of methyl groups of acetate was allowed to refine subject to geometric restraints.

### Crystal data

**Table:**

[Zn_4_(C_19_H_20_N_2_O_4_)_2_(C_2_H_3_O_2_)_4_]	*Z* = 1
*M**_r_* = 1178.48	*F*(000) = 604
Triclinic, *P*1	*D*_x_ = 1.684 Mg m^−^^3^
Hall symbol: -P 1	Mo *K*α radiation, λ = 0.71073 Å
*a* = 10.4894 (9) Å	Cell parameters from 4019 reflections
*b* = 10.7917 (9) Å	θ = 2.2–27.5°
*c* = 11.9550 (11) Å	µ = 2.12 mm^−^^1^
α = 103.425 (2)°	*T* = 298 K
β = 94.323 (1)°	Block, yellow
γ = 115.677 (1)°	0.15 × 0.10 × 0.08 mm
*V* = 1162.17 (18) Å^3^	

### Data collection

**Table:**

Bruker SMART APEX CCD area-detector diffractometer	4497 independent reflections
Radiation source: fine-focus sealed tube	3969 reflections with *I* > 2σ(*I*)
Graphite monochromator	*R*_int_ = 0.013
Detector resolution: 0 pixels mm^-1^	θ_max_ = 26.0°, θ_min_ = 1.8°
phi and ω scans	*h* = −6→12
Absorption correction: multi-scan (*SADABS*; Sheldrick, 1996)	*k* = −13→13
*T*_min_ = 0.742, *T*_max_ = 0.849	*l* = −13→14
6281 measured reflections	

### Refinement

**Table:**

Refinement on *F*^2^	Primary atom site location: structure-invariant direct methods
Least-squares matrix: full	Secondary atom site location: difference Fourier map
*R*[*F*^2^ > 2σ(*F*^2^)] = 0.024	Hydrogen site location: inferred from neighbouring sites
*wR*(*F*^2^) = 0.071	H-atom parameters constrained
*S* = 1.05	*w* = 1/[σ^2^(*F*_o_^2^) + (0.0367*P*)^2^ + 0.3975*P*] where *P* = (*F*_o_^2^ + 2*F*_c_^2^)/3
4497 reflections	(Δ/σ)_max_ = 0.006
318 parameters	Δρ_max_ = 0.26 e Å^−^^3^
0 restraints	Δρ_min_ = −0.27 e Å^−^^3^

### Special details

**Table:**

Geometry. All e.s.d.'s (except the e.s.d. in the dihedral angle between two l.s. planes) are estimated using the full covariance matrix. The cell e.s.d.'s are taken into account individually in the estimation of e.s.d.'s in distances, angles and torsion angles; correlations between e.s.d.'s in cell parameters are only used when they are defined by crystal symmetry. An approximate (isotropic) treatment of cell e.s.d.'s is used for estimating e.s.d.'s involving l.s. planes.
Refinement. Refinement of *F*^2^ against ALL reflections. The weighted *R*-factor *wR* and goodness of fit *S* are based on *F*^2^, conventional *R*-factors *R* are based on *F*, with *F* set to zero for negative *F*^2^. The threshold expression of *F*^2^ > σ(*F*^2^) is used only for calculating *R*-factors(gt) *etc*. and is not relevant to the choice of reflections for refinement. *R*-factors based on *F*^2^ are statistically about twice as large as those based on *F*, and *R*- factors based on ALL data will be even larger.

### Fractional atomic coordinates and isotropic or equivalent isotropic displacement parameters (Å^2^)

**Table:**

	*x*	*y*	*z*	*U*_iso_*/*U*_eq_	
C1	0.5747 (2)	0.1717 (3)	0.1390 (2)	0.0479 (6)	
H1A	0.5651	0.0938	0.1692	0.072*	
H1B	0.5348	0.1350	0.0563	0.072*	
H1C	0.5239	0.2186	0.1783	0.072*	
C2	0.7601 (2)	0.3909 (2)	0.12186 (18)	0.0334 (4)	
C3	0.6657 (2)	0.4234 (3)	0.0636 (2)	0.0431 (5)	
H3	0.5666	0.3642	0.0497	0.052*	
C4	0.7206 (3)	0.5459 (3)	0.0258 (2)	0.0540 (7)	
H4	0.6577	0.5680	−0.0144	0.065*	
C5	0.8656 (3)	0.6339 (3)	0.0473 (2)	0.0485 (6)	
H5	0.9005	0.7136	0.0193	0.058*	
C6	0.9640 (2)	0.6057 (2)	0.11148 (18)	0.0344 (5)	
C7	0.9110 (2)	0.4824 (2)	0.14919 (17)	0.0288 (4)	
C8	1.1149 (2)	0.7072 (2)	0.13321 (19)	0.0374 (5)	
H8	1.1384	0.7791	0.0964	0.045*	
C9	1.3647 (2)	0.8311 (2)	0.2054 (2)	0.0428 (5)	
H9A	1.3563	0.9191	0.2172	0.051*	
H9B	1.3959	0.8132	0.1316	0.051*	
C10	1.4783 (2)	0.8521 (2)	0.3040 (2)	0.0438 (5)	
H10A	1.4411	0.8567	0.3759	0.053*	
H10B	1.5627	0.9436	0.3152	0.053*	
C11	1.5238 (3)	0.7346 (3)	0.2830 (2)	0.0503 (6)	
H11A	1.5415	0.7156	0.2039	0.060*	
H11B	1.6138	0.7683	0.3370	0.060*	
C12	1.4643 (2)	0.5208 (3)	0.33241 (19)	0.0396 (5)	
H12	1.5642	0.5583	0.3466	0.048*	
C13	1.3848 (2)	0.3817 (2)	0.35174 (18)	0.0362 (5)	
C14	1.4677 (3)	0.3186 (3)	0.3880 (2)	0.0446 (6)	
H14	1.5679	0.3689	0.4015	0.053*	
C15	1.4038 (3)	0.1858 (3)	0.4035 (2)	0.0499 (6)	
H15	1.4602	0.1465	0.4280	0.060*	
C16	1.2532 (3)	0.1083 (3)	0.3828 (2)	0.0435 (5)	
H16	1.2093	0.0169	0.3925	0.052*	
C17	1.1699 (2)	0.1679 (2)	0.34775 (18)	0.0354 (5)	
C18	1.2338 (2)	0.3069 (2)	0.33311 (17)	0.0310 (4)	
C19	0.9443 (3)	−0.0457 (3)	0.3169 (3)	0.0661 (8)	
H19A	0.8425	−0.0761	0.2991	0.099*	
H19B	0.9686	−0.0585	0.3910	0.099*	
H19C	0.9691	−0.1021	0.2566	0.099*	
C20	0.7933 (2)	−0.0082 (2)	0.06099 (19)	0.0343 (4)	
C21	0.7849 (2)	−0.1000 (3)	−0.0585 (2)	0.0422 (5)	
H21A	0.7129	−0.1974	−0.0704	0.063*	
H21B	0.8769	−0.0973	−0.0633	0.063*	
H21C	0.7594	−0.0635	−0.1179	0.063*	
C22	0.8649 (2)	0.3057 (2)	0.48569 (18)	0.0314 (4)	
C23	1.0099 (3)	0.3370 (3)	0.5482 (2)	0.0493 (6)	
H23A	1.0778	0.4354	0.5584	0.074*	
H23B	1.0424	0.2747	0.5026	0.074*	
H23C	1.0022	0.3208	0.6236	0.074*	
N1	1.22093 (19)	0.71044 (18)	0.19729 (15)	0.0340 (4)	
N2	1.41504 (19)	0.59888 (19)	0.29835 (16)	0.0359 (4)	
O1	0.99395 (15)	0.44590 (15)	0.20882 (13)	0.0329 (3)	
O2	1.14934 (15)	0.35966 (15)	0.30055 (13)	0.0343 (3)	
O3	0.72225 (16)	0.27149 (18)	0.15849 (17)	0.0488 (4)	
O4	1.02202 (17)	0.10166 (16)	0.32310 (15)	0.0437 (4)	
O5	0.89752 (18)	0.11959 (17)	0.09127 (14)	0.0436 (4)	
O6	0.7050 (2)	−0.0567 (2)	0.12112 (17)	0.0590 (5)	
O7	0.82953 (16)	0.25334 (17)	0.37574 (13)	0.0409 (4)	
O8	0.77997 (17)	0.33076 (18)	0.54407 (13)	0.0423 (4)	
Zn1	1.20447 (2)	0.56385 (2)	0.28798 (2)	0.02906 (8)	
Zn2	0.92824 (2)	0.25063 (2)	0.24271 (2)	0.02988 (8)	

### Atomic displacement parameters (Å^2^)

**Table:**

	*U*^11^	*U*^22^	*U*^33^	*U*^12^	*U*^13^	*U*^23^
C1	0.0285 (12)	0.0518 (14)	0.0543 (15)	0.0087 (11)	0.0080 (10)	0.0197 (12)
C2	0.0322 (11)	0.0350 (11)	0.0342 (11)	0.0155 (9)	0.0064 (9)	0.0125 (9)
C3	0.0314 (12)	0.0494 (13)	0.0485 (13)	0.0184 (11)	0.0017 (10)	0.0174 (11)
C4	0.0471 (15)	0.0605 (16)	0.0624 (17)	0.0293 (13)	−0.0030 (12)	0.0281 (14)
C5	0.0517 (15)	0.0470 (14)	0.0532 (15)	0.0243 (12)	0.0024 (12)	0.0258 (12)
C6	0.0383 (12)	0.0344 (11)	0.0332 (11)	0.0177 (9)	0.0062 (9)	0.0136 (9)
C7	0.0311 (10)	0.0288 (10)	0.0270 (10)	0.0145 (8)	0.0052 (8)	0.0081 (8)
C8	0.0461 (13)	0.0308 (11)	0.0363 (11)	0.0151 (10)	0.0110 (10)	0.0164 (9)
C9	0.0401 (13)	0.0356 (12)	0.0433 (13)	0.0061 (10)	0.0100 (10)	0.0178 (10)
C10	0.0331 (12)	0.0387 (12)	0.0445 (13)	0.0024 (10)	0.0072 (10)	0.0141 (10)
C11	0.0302 (12)	0.0540 (15)	0.0619 (16)	0.0099 (11)	0.0159 (11)	0.0262 (13)
C12	0.0259 (11)	0.0473 (13)	0.0384 (12)	0.0149 (10)	0.0045 (9)	0.0046 (10)
C13	0.0352 (12)	0.0427 (12)	0.0292 (10)	0.0220 (10)	0.0001 (8)	0.0019 (9)
C14	0.0394 (13)	0.0543 (14)	0.0402 (12)	0.0284 (12)	−0.0019 (10)	0.0041 (11)
C15	0.0579 (16)	0.0616 (16)	0.0421 (13)	0.0432 (14)	−0.0025 (11)	0.0093 (11)
C16	0.0597 (16)	0.0434 (13)	0.0363 (12)	0.0326 (12)	0.0053 (11)	0.0113 (10)
C17	0.0424 (12)	0.0379 (11)	0.0291 (10)	0.0226 (10)	0.0064 (9)	0.0077 (9)
C18	0.0332 (11)	0.0353 (11)	0.0254 (10)	0.0193 (9)	0.0029 (8)	0.0048 (8)
C19	0.0645 (19)	0.0417 (15)	0.085 (2)	0.0146 (14)	0.0019 (16)	0.0313 (15)
C20	0.0313 (11)	0.0406 (12)	0.0349 (11)	0.0190 (10)	0.0052 (9)	0.0134 (9)
C21	0.0367 (12)	0.0430 (12)	0.0406 (12)	0.0185 (10)	0.0017 (10)	0.0035 (10)
C22	0.0355 (11)	0.0279 (10)	0.0316 (10)	0.0131 (9)	0.0107 (9)	0.0121 (8)
C23	0.0518 (15)	0.0672 (16)	0.0374 (12)	0.0374 (13)	0.0075 (11)	0.0111 (12)
N1	0.0332 (10)	0.0307 (9)	0.0325 (9)	0.0083 (8)	0.0084 (7)	0.0119 (7)
N2	0.0279 (9)	0.0393 (10)	0.0364 (10)	0.0125 (8)	0.0083 (7)	0.0100 (8)
O1	0.0255 (7)	0.0312 (7)	0.0399 (8)	0.0094 (6)	0.0001 (6)	0.0163 (6)
O2	0.0269 (7)	0.0315 (7)	0.0460 (9)	0.0138 (6)	0.0035 (6)	0.0149 (6)
O3	0.0254 (8)	0.0468 (9)	0.0741 (12)	0.0089 (7)	0.0038 (8)	0.0355 (9)
O4	0.0397 (9)	0.0345 (8)	0.0589 (10)	0.0168 (7)	0.0095 (8)	0.0182 (7)
O5	0.0423 (9)	0.0382 (9)	0.0384 (9)	0.0129 (7)	0.0064 (7)	0.0024 (7)
O6	0.0571 (11)	0.0699 (12)	0.0544 (11)	0.0260 (10)	0.0266 (9)	0.0283 (10)
O7	0.0369 (8)	0.0534 (9)	0.0281 (8)	0.0185 (7)	0.0093 (6)	0.0086 (7)
O8	0.0365 (9)	0.0551 (10)	0.0323 (8)	0.0206 (8)	0.0113 (7)	0.0077 (7)
Zn1	0.02476 (13)	0.02968 (13)	0.03096 (13)	0.00999 (10)	0.00664 (9)	0.01083 (10)
Zn2	0.02869 (14)	0.02835 (13)	0.02989 (13)	0.01035 (10)	0.00723 (10)	0.00921 (10)

### Geometric parameters (Å, º)

**Table:**

C1—O3	1.412 (3)	C15—C16	1.398 (4)
C1—H1A	0.9600	C15—H15	0.9300
C1—H1B	0.9600	C16—C17	1.382 (3)
C1—H1C	0.9600	C16—H16	0.9300
C2—O3	1.362 (3)	C17—O4	1.370 (3)
C2—C3	1.378 (3)	C17—C18	1.414 (3)
C2—C7	1.416 (3)	C18—O2	1.322 (2)
C3—C4	1.394 (3)	C19—O4	1.418 (3)
C3—H3	0.9300	C19—H19A	0.9600
C4—C5	1.363 (4)	C19—H19B	0.9600
C4—H4	0.9300	C19—H19C	0.9600
C5—C6	1.417 (3)	C20—O6	1.226 (3)
C5—H5	0.9300	C20—O5	1.277 (3)
C6—C7	1.399 (3)	C20—C21	1.509 (3)
C6—C8	1.441 (3)	C21—H21A	0.9600
C7—O1	1.325 (2)	C21—H21B	0.9600
C8—N1	1.285 (3)	C21—H21C	0.9600
C8—H8	0.9300	C22—O8	1.253 (2)
C9—N1	1.482 (3)	C22—O7	1.259 (2)
C9—C10	1.514 (3)	C22—C23	1.501 (3)
C9—H9A	0.9700	C23—H23A	0.9600
C9—H9B	0.9700	C23—H23B	0.9600
C10—C11	1.515 (4)	C23—H23C	0.9600
C10—H10A	0.9700	N1—Zn1	2.0727 (17)
C10—H10B	0.9700	N2—Zn1	2.0616 (18)
C11—N2	1.479 (3)	O1—Zn1	2.0203 (14)
C11—H11A	0.9700	O1—Zn2	2.0639 (14)
C11—H11B	0.9700	O2—Zn2	2.0632 (14)
C12—N2	1.284 (3)	O2—Zn1	2.0676 (14)
C12—C13	1.452 (3)	O3—Zn2	2.4356 (16)
C12—H12	0.9300	O5—Zn2	1.9261 (15)
C13—C18	1.402 (3)	O7—Zn2	1.9635 (14)
C13—C14	1.415 (3)	O8—Zn1^i^	2.0201 (15)
C14—C15	1.359 (4)	Zn1—O8^i^	2.0201 (15)
C14—H14	0.9300		
			
O3—C1—H1A	109.5	C13—C18—C17	118.12 (19)
O3—C1—H1B	109.5	O4—C19—H19A	109.5
H1A—C1—H1B	109.5	O4—C19—H19B	109.5
O3—C1—H1C	109.5	H19A—C19—H19B	109.5
H1A—C1—H1C	109.5	O4—C19—H19C	109.5
H1B—C1—H1C	109.5	H19A—C19—H19C	109.5
O3—C2—C3	125.4 (2)	H19B—C19—H19C	109.5
O3—C2—C7	112.88 (18)	O6—C20—O5	124.8 (2)
C3—C2—C7	121.7 (2)	O6—C20—C21	120.8 (2)
C2—C3—C4	119.1 (2)	O5—C20—C21	114.42 (19)
C2—C3—H3	120.4	C20—C21—H21A	109.5
C4—C3—H3	120.4	C20—C21—H21B	109.5
C5—C4—C3	120.6 (2)	H21A—C21—H21B	109.5
C5—C4—H4	119.7	C20—C21—H21C	109.5
C3—C4—H4	119.7	H21A—C21—H21C	109.5
C4—C5—C6	121.0 (2)	H21B—C21—H21C	109.5
C4—C5—H5	119.5	O8—C22—O7	120.8 (2)
C6—C5—H5	119.5	O8—C22—C23	119.36 (19)
C7—C6—C5	119.1 (2)	O7—C22—C23	119.80 (19)
C7—C6—C8	123.83 (19)	C22—C23—H23A	109.5
C5—C6—C8	117.1 (2)	C22—C23—H23B	109.5
O1—C7—C6	123.77 (18)	H23A—C23—H23B	109.5
O1—C7—C2	117.92 (17)	C22—C23—H23C	109.5
C6—C7—C2	118.31 (18)	H23A—C23—H23C	109.5
N1—C8—C6	127.81 (19)	H23B—C23—H23C	109.5
N1—C8—H8	116.1	C8—N1—C9	115.46 (18)
C6—C8—H8	116.1	C8—N1—Zn1	125.35 (15)
N1—C9—C10	113.12 (18)	C9—N1—Zn1	119.19 (14)
N1—C9—H9A	109.0	C12—N2—C11	116.01 (19)
C10—C9—H9A	109.0	C12—N2—Zn1	124.81 (15)
N1—C9—H9B	109.0	C11—N2—Zn1	118.53 (15)
C10—C9—H9B	109.0	C7—O1—Zn1	129.06 (12)
H9A—C9—H9B	107.8	C7—O1—Zn2	125.64 (12)
C9—C10—C11	114.1 (2)	Zn1—O1—Zn2	105.29 (6)
C9—C10—H10A	108.7	C18—O2—Zn2	127.10 (13)
C11—C10—H10A	108.7	C18—O2—Zn1	129.24 (13)
C9—C10—H10B	108.7	Zn2—O2—Zn1	103.62 (6)
C11—C10—H10B	108.7	C2—O3—C1	119.07 (18)
H10A—C10—H10B	107.6	C2—O3—Zn2	113.52 (12)
N2—C11—C10	113.20 (19)	C1—O3—Zn2	127.31 (14)
N2—C11—H11A	108.9	C17—O4—C19	119.41 (19)
C10—C11—H11A	108.9	C20—O5—Zn2	120.25 (14)
N2—C11—H11B	108.9	C22—O7—Zn2	137.14 (14)
C10—C11—H11B	108.9	C22—O8—Zn1^i^	135.32 (15)
H11A—C11—H11B	107.8	O1—Zn1—O8^i^	109.18 (6)
N2—C12—C13	128.7 (2)	O1—Zn1—N2	149.75 (7)
N2—C12—H12	115.7	O8^i^—Zn1—N2	98.57 (7)
C13—C12—H12	115.7	O1—Zn1—O2	75.35 (5)
C18—C13—C14	119.5 (2)	O8^i^—Zn1—O2	100.83 (6)
C18—C13—C12	123.98 (19)	N2—Zn1—O2	88.08 (6)
C14—C13—C12	116.5 (2)	O1—Zn1—N1	88.64 (6)
C15—C14—C13	121.3 (2)	O8^i^—Zn1—N1	103.96 (7)
C15—C14—H14	119.3	N2—Zn1—N1	96.30 (7)
C13—C14—H14	119.3	O2—Zn1—N1	153.84 (7)
C14—C15—C16	120.0 (2)	O5—Zn2—O7	137.44 (7)
C14—C15—H15	120.0	O5—Zn2—O2	104.92 (7)
C16—C15—H15	120.0	O7—Zn2—O2	110.70 (6)
C17—C16—C15	119.8 (2)	O5—Zn2—O1	103.31 (7)
C17—C16—H16	120.1	O7—Zn2—O1	107.96 (6)
C15—C16—H16	120.1	O2—Zn2—O1	74.53 (5)
O4—C17—C16	125.1 (2)	O5—Zn2—O3	85.68 (7)
O4—C17—C18	113.64 (18)	O7—Zn2—O3	79.46 (6)
C16—C17—C18	121.3 (2)	O2—Zn2—O3	143.50 (5)
O2—C18—C13	122.92 (19)	O1—Zn2—O3	69.03 (5)
O2—C18—C17	118.95 (19)		

Symmetry code: (i) −*x*+2, −*y*+1, −*z*+1.
