# Supplementary figures and images for: Crystal structure of di-μ-acetato-di­acetatobis­(μ-6,6′-dimeth­oxy-2,2′-{[(propane-1,3-diylbis(aza­nylyl­idene)]bis­(methanylyl­idene)}diphenolato)tetra­zinc
Source: Acta Crystallogr E Crystallogr Commun. 2015 Nov 11;71(Pt 12):m217–8. doi: 10.1107/S2056989015020551 (PMC4719841; doi:10.1107/S2056989015020551)

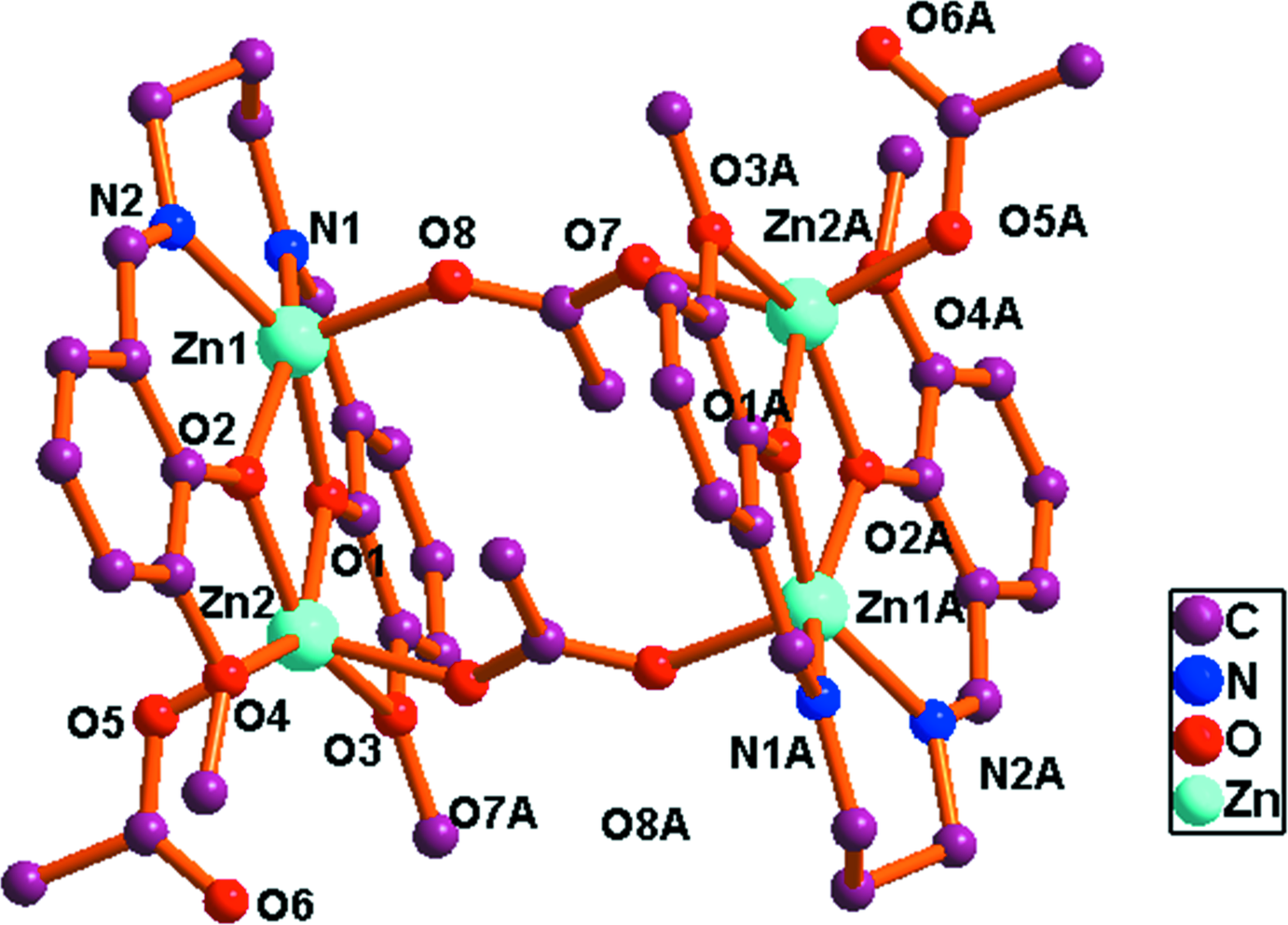

Supplement: Supplementary file 3 [file e-71-0m217-fig1.tif]

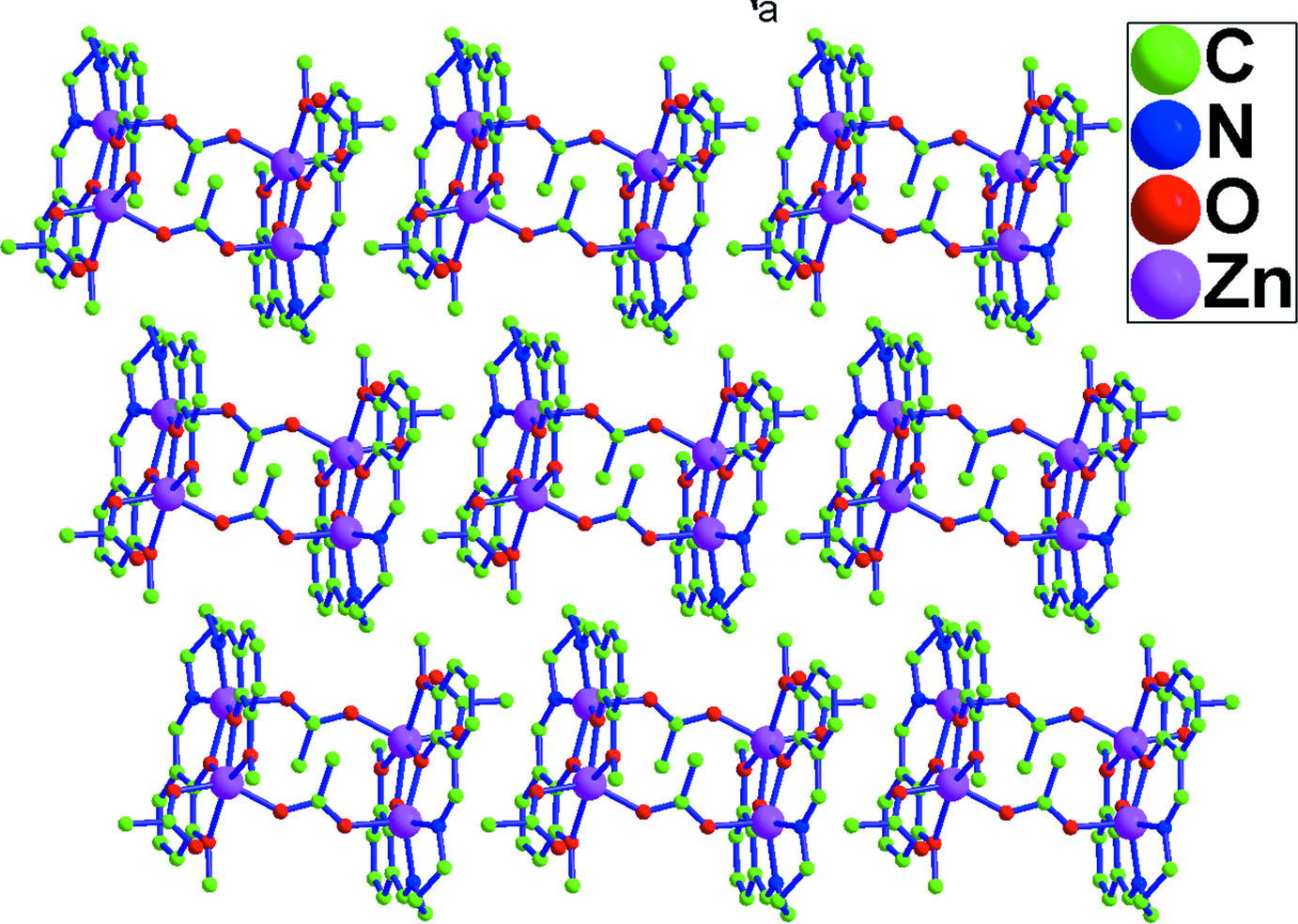

Supplement: Supplementary file 4 [file e-71-0m217-fig2.tif]
